# Limited Cutaneous Scleroderma: A Case Report

**DOI:** 10.7759/cureus.45336

**Published:** 2023-09-16

**Authors:** Manvi Marathe, Shweta Borkar

**Affiliations:** 1 Medicine, Jawaharlal Nehru Medical College, Datta Meghe Institute of Higher Education and Research, Wardha, IND; 2 Internal Medicine, Datta Meghe Medical College, Nagpur, IND

**Keywords:** crest syndrome, vitiligo, cutaneous sclerosis, morphea, scleroderma

## Abstract

Scleroderma is an uncommon disease that affects the connective tissue, causing skin hardening and sometimes organ damage. There are two main forms of scleroderma: localised scleroderma, or morphea, which usually has a mild and limited course and only affects the skin and/or the tissues below it, and systemic sclerosis, which involves skin hardening and internal organ problems. The cause of localised scleroderma is unknown. Recent studies suggest that this form can have different levels of severity and can affect some organs. To avoid complications due to the high morbidity of localised scleroderma, early treatment is recommended. In this article, we present the main aspects and details of the management of patients with localised scleroderma.

## Introduction

Scleroderma is a disease that affects the connective tissue, making the skin stiff and sometimes harming the organs. There are two main kinds of scleroderma: localised scleroderma (LoS), or morphea, which is usually mild and limited and only involves the skin and/or the underlying tissues; and systemic sclerosis (SSc), which causes skin hardening and organ problems (especially the oesophagus, lung, and vascular system). Chronic connective tissue disease with no known cause is characterised as LoS, often known as morphea [1]. There are various forms of morphea, and each has a unique clinical presentation and degree of connective tissue involvement. Skin thickening and increased collagen production in the indurative lesion are the hallmarks of morphea. Linear scleroderma, plaque morphea, deep morphea, bullous morphea, and generalised morphea are the different subtypes of this entity. The severity of each of these kinds' effects on the face can vary. The most typical form of infantile scleroderma is LoS. LoS classifications are not mutually exclusive because multiple subtypes might coexist in a single patient [2,3]. LoS is a rare illness with an annual incidence of between 0.3 and three cases per 100,000 people. With 2-4 women to one male, it is more prevalent among Caucasian women. Children and adults both have equal prevalence rates. Ninety percent of children are diagnosed between the ages of two and 14 while the highest incidence in adults occurs in the fifth decade of life. According to the literature, LoS is not just a cutaneous condition. Particularly in adults with the localised form of the disease, there is evidence of internal organ involvement, relationship with other connective tissue diseases, and uncommon transitional forms for SSc [4].

## Case presentation

**Figure 1:**
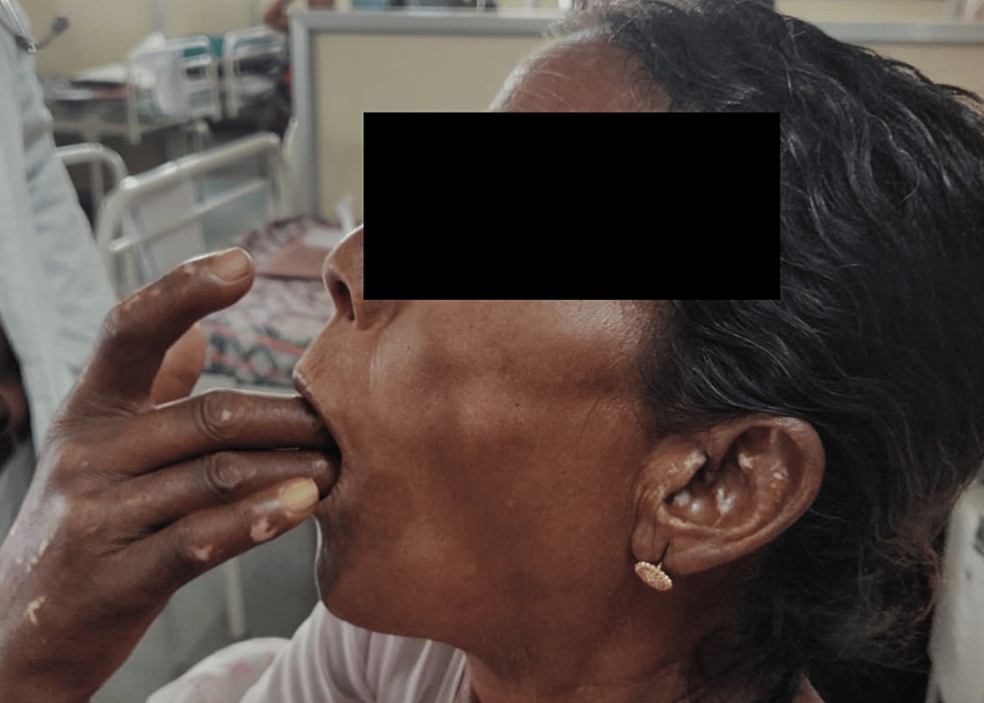
Image Showing Reduced Mouth Opening

**Figure 2:**
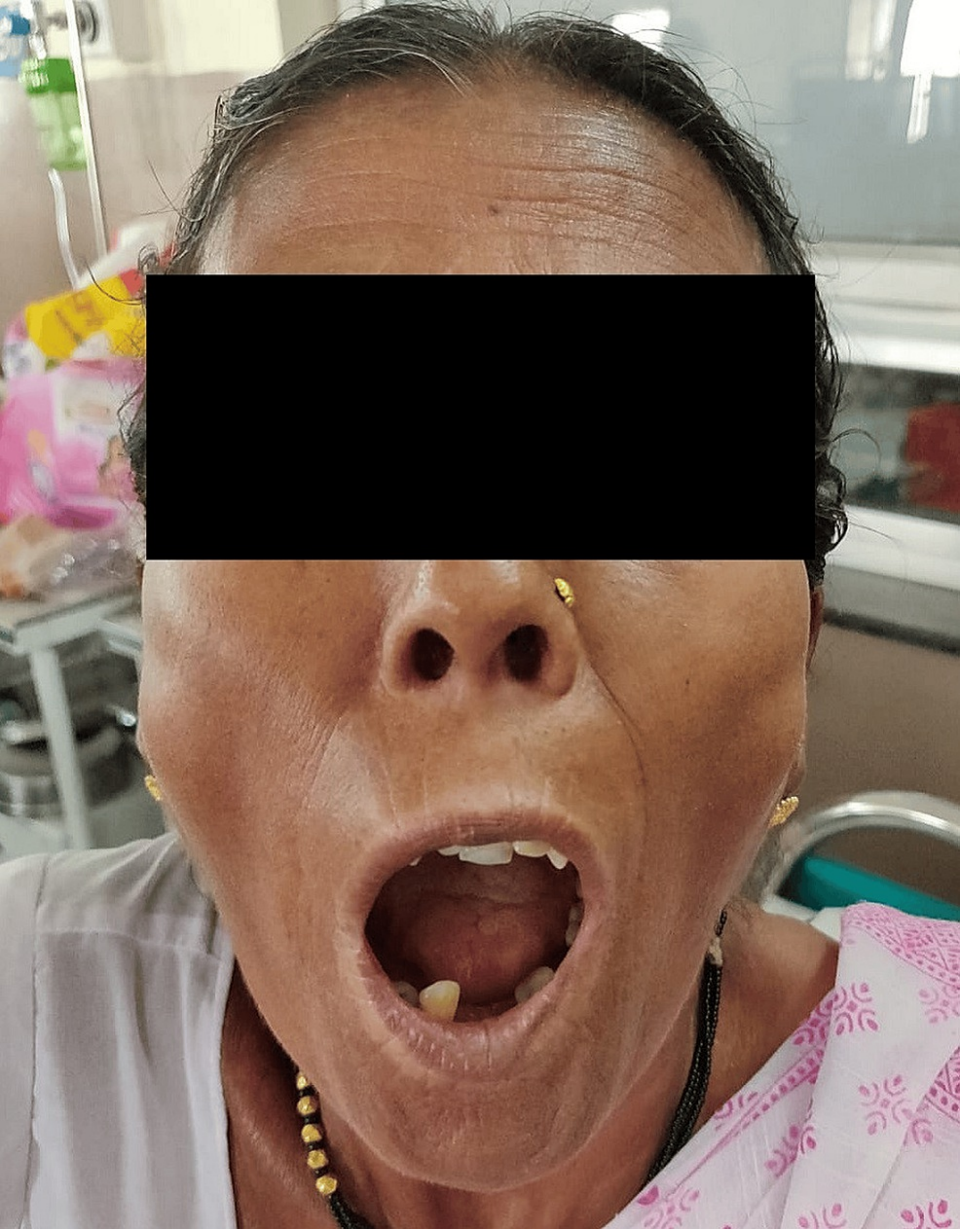
Image Showing Fish Mouth Appearance

**Figure 3:**
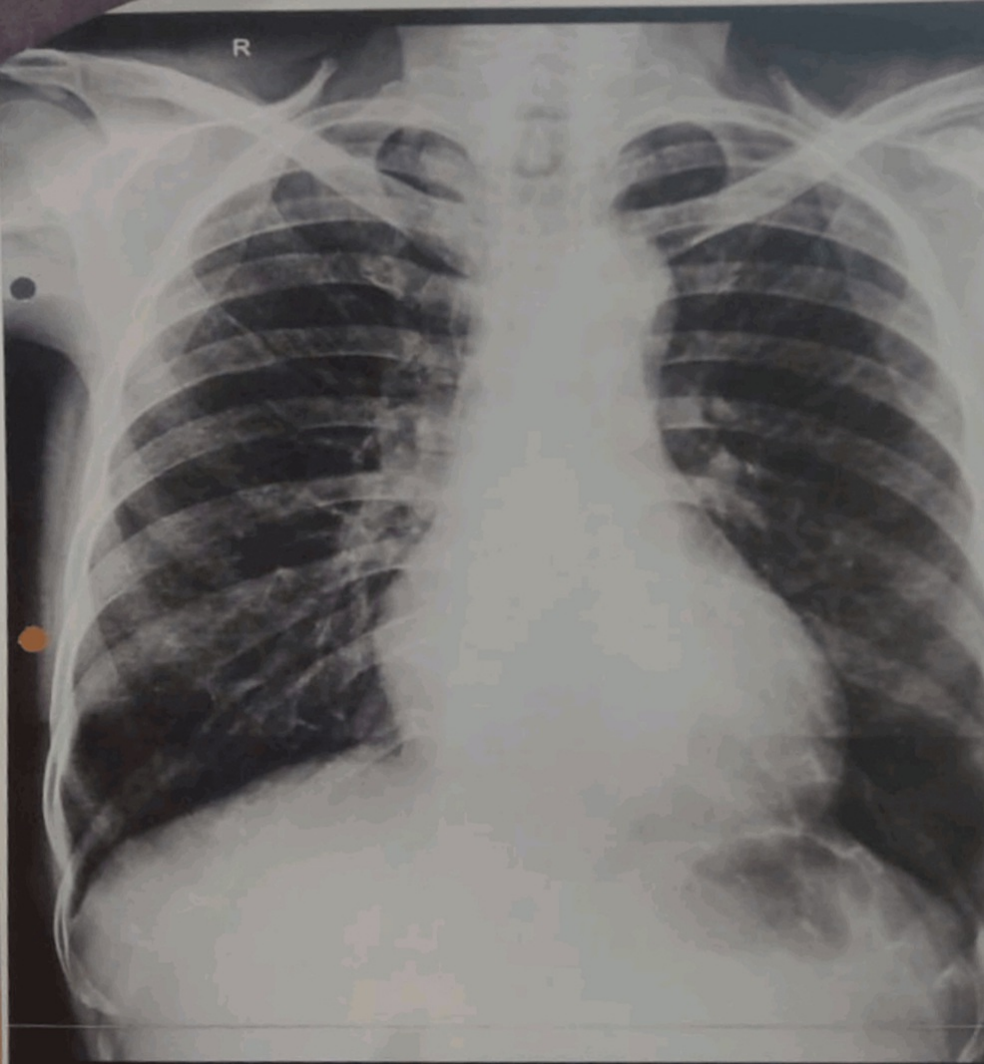
Chest X-ray Showing No Systemic Involvement

**Figure 4:**
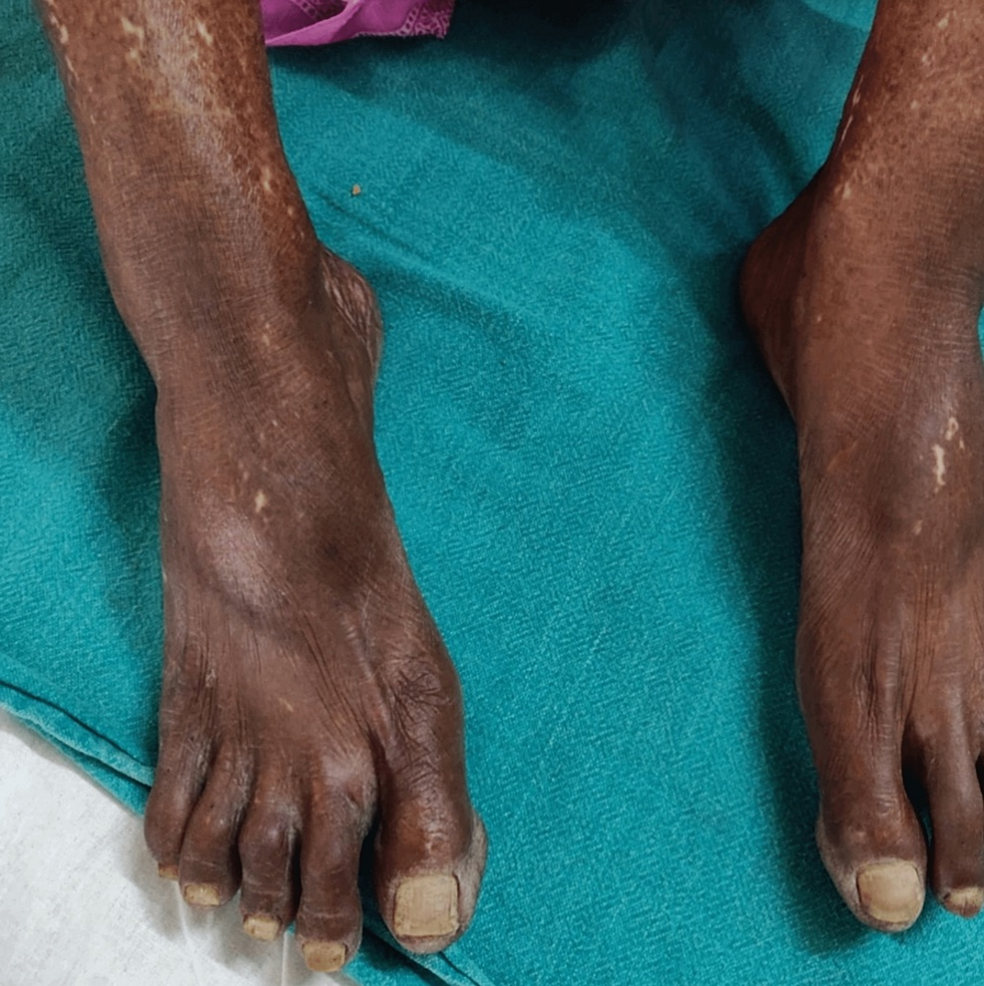
Image Showing Skin Tightening with Shiny Appearance

A 60-year-old hypertensive woman with an unremarkable medical and surgical history with no associated co-morbidities presented to our institute with joint stiffness, burning sensation, and generalised weakness all over the body. She was given symptomatic treatment which is ibuprofen and omeprazole, basic investigations were done. She was advised to an electrocardiogram (ECG), chest X-ray and ultra-sound sonography test (USG) (abdomen and pelvis). All the reports were normal. Hb%: 11.5g/dL; RBC count: 3.83million/mm^3^; WBC count: 7900/mm^3^; platelet count: 2.48/mcL. Serum creatinine: 0.61mg/dL; serum potassium: 3.13mEq/L; serum sodium: 144 mmol/L; serum urea: 18.45mg/dL. Albumin: 3.45g/dL; alkaline phosphatase: 70.56U/L; alanine transaminase (ALT): 15.64U/L; aspartate aminotransferase (AST): 28.07U/L; bilirubin (total): 0.42mg/dL; HDL (cholesterol): 34.34mg/dL; cholesterol total: 157.46mg/dL; LDL (low-density lipoprotein) cholesterol: 98mg/dL; triglyceride: 122.94mg/dL; C-reactive protein (CRP): 0.3mg/dL; erythrocyte sedimentation rate (ESR): 18mm/hr. The clinical features and diagnostic tests were compatible with limited cutaneous scleroderma; antinuclear antibody: positive; rheumatoid factor: positive; anti-U1-riboprotein (RNP): positive; anti-centromere antibody: negative. The patient showed reduced mouth opening and fish mouth appearance (Figures 1, 2). A chest X-ray was done to check for systemic involvement and was found out to be normal (Figure 3). The patient also had skin tightening and features of vitiligo that is salt and pepper lesions typical of SSc (Figure 4).

She was started with methotrexate, steroids and physiotherapy to limit the progression. The patient showed no signs of systemic involvement. All investigations were done to check for the same. The patient was advised regular follow-ups every three months to check for progression and for early diagnosis of extra-cutaneous/systemic manifestations.

## Discussion

The course of SSc throughout is with limited cutaneous involvement. We classify systemic SSc a category of disorders known as SSc, which has a diverse range of clinical and laboratory symptoms. The best way to understand its natural history is to categorise the two main clinical types into early and late phases and monitor changes in skin thickness and other disease signs throughout time [5]. The skin is either normal or only thickened in the hands and feet (not above the elbows or knees). Erosis with widespread skin involvement is classified as early (less than three years) or late (more than six years). SSc with limited skin involvement is classified as early (less than five years) or late (more than 10 years). The disease duration is measured from the first symptom that is related to SSc. Limited SSc usually starts with Raynaud’s phenomenon, affects the skin below the elbows, causes problems in the digestive and respiratory systems, and is associated with antibodies against the centromere [6]. Classification of the disease is not simple due to its multiple presentations [7]. LoS is the most common form in children and is mostly restricted to the skin [8]. Generally, LoS is divided into the following types: plaque morphea, generalised morphea, bullous morphea, and linear scleroderma (Table 1).

**Table 1:** Types of Localised Scleroderma

Types of Localised Scleroderma
Plaque Morphea
Generalised Morphea
Bullous Morphea
Linear Morphea

Including subtypes that involve the head and face, linear scleroderma ‘en coup de sabre' (LScs), progressive facial hemiatrophy (PFH), and deep morphea. In this case, the patient has reduced mouth opening with a fish mouth appearance as the skin develops a diffuse, hard texture, tightening of soft tissues of face and mouth, with no systemic involvement.

Extracutaneous manifestations

Systemic symptoms, which often appear a few months after the onset of LoS, frequently appear before skin disease. The disease typically affects the skin's outermost layers, which harden and thicken [9]. LoS can result in significant impairment due to joint contractures and subcutaneous atrophy, although never being lethal [10]. Extracutaneous involvements in patients with LoS are described as including Raynaud's phenomenon, localised hair loss at the affected site, ocular involvement, neurological involvement, gastroesophageal reflux disease (GERD), esophagitis (documented by upper gastrointestinal endoscopy), abnormal pulmonary function testing (PFP), restrictive lung disease, cough or dyspnea, and fascia or muscle involvement. There is also documentation of dental changes. Atrophy of the tongue and malocclusions may result from these alterations. The neurological issue that scleroderma is most usually associated with is complex partial seizures. Ocular issues fall into four categories: adnexal structural involvement, anterior segment involvement, posterior segment involvement, and ocular central nervous system (CNS) involvement. In general, the likelihood of developing a related visceral anomaly increases with the length and depth of the sclerodermiform process. A 0.9 to 1.3% prevalence of linear scleroderma is evolving into SSc [11,12].

Management

It has been advised to employ topical drugs, immunosuppressive pharmaceuticals, physical therapy, and phototherapy, among other therapeutic approaches. LoS can cause problems with movement and appearance, so it is important to start treatment as soon as possible to prevent complications. Treatment can help prevent new or bigger skin lesions from forming. Some of the possible treatment options for LoS are D-penicillamine, vitamin D (topical or oral), psoralen-UVA light therapy, phenytoin, corticosteroids, methotrexate, cyclosporine, and interferon. Corticosteroids can be given orally or by injection, alone or with methotrexate. The usual dose of methotrexate is 1mg/kg/week by injection under the skin, up to a maximum of 25mg/week. Folic acid should also be taken daily (0.4-1 mg) or weekly (5 mg) to reduce side effects. The most effective treatments are methotrexate with or without corticosteroids for severe disease that affects more than the skin and ultraviolet A1 light therapy for mild disease that only affects the skin. The employment of physiatry and plastic surgery methods is another treatment choice. Patients with joint contractures and restricted extremity motion due to morphea receive physical treatment [13-15].

## Conclusions

In this case report, we presented a case of limited scleroderma. The clinical features and diagnostic tests were confirmative of the same. The patient had no systemic involvement. The approach towards the treatment and an attempt to improve the prognosis was made by starting corticosteroids and methotrexate therapy. The patient was advised regular follow-ups every three months to check for progression and for early diagnosis of extra-cutaneous/systemic manifestations.
